# Supply-side factors influencing demand for facility-based delivery in Tanzania: a multilevel analysis

**DOI:** 10.1186/s13561-023-00468-1

**Published:** 2023-11-06

**Authors:** Peter Binyaruka, Anna Foss, Abdullah Alibrahim, Nicholaus Mziray, Rachel Cassidy, Josephine Borghi

**Affiliations:** 1 Department of Health System, Impact Evaluation and Policy, Ifakara Health Institute, PO Box 78373, Dar es Salaam, Tanzania; 2 Department of Global Health and Development, London School of Hygiene and Tropical Medicine, 15-17 Tavistock Place, London, WC1H 9SH UK; 3 College of Engineering and Petroleum, Kuwait University, Kuwait City, Kuwait; 4 KPM Center for Public Management, University of Bern, Schanzeneckstrasse 1, Bern, 3012 Switzerland

**Keywords:** Demand, Supply, Service utilisation, Facility birth, Delivery care, Tanzania

## Abstract

**Background:**

Improving access to facility-based delivery care has the potential to reduce maternal and newborn deaths across settings. Yet, the access to a health facility for childbirth remains low especially in low-income settings. To inform evidence-based interventions, more evidence is needed especially accounting for demand- and supply-side factors influencing access to facility-based delivery care. We aimed to fill this knowledge gap using data from Tanzania.

**Methods:**

We used data from a cross-sectional survey (conducted in January 2012) of 150 health facilities, 1494 patients and 2846 households with women who had given births in the last 12 months before the survey across 11 districts in three regions in Tanzania. The main outcome was the place of delivery (giving birth in a health facility or otherwise), while explanatory variables were measured at the individual woman and facility level. Given the hierarchical structure of the data and variance in demand across facilities, we used a multilevel mixed-effect logistic regression to explore the determinants of facility-based delivery care.

**Results:**

Eighty-six percent of 2846 women gave birth in a health facility. Demand for facility-based delivery care was influenced more by demand-side factors (76%) than supply-side factors (24%). On demand-side factors, facility births were more common among women who were educated, Muslim, wealthier, with their first childbirth, and those who had at least four antenatal care visits. On supply-side factors, facility births were more common in facilities offering outreach services, longer consultation times and higher interpersonal quality. In contrast, facilities with longer average waiting times, longer travel times and higher chances of charging delivery fees had few facility births.

**Conclusions:**

Policy responses should aim for strategies to improve demand like health education to raise awareness towards care seeking among less educated groups and those with higher parity, reduce financial barriers to access (including time costs to reach and access care), and policy interventions to enhance interpersonal quality in service provision.

**Supplementary Information:**

The online version contains supplementary material available at 10.1186/s13561-023-00468-1.

## Introduction

Maternal mortality remains a global health challenge [1, 2]. Overall, the global maternal mortality rate (MMR) in 2020 was estimated at 223 maternal deaths per 100 000 live births [1, 2], substantially higher than the Sustainable Development Goal (SDG) three target of 70 maternal deaths per 100,000 live births by 2030 [3]. Although the MMR has dropped by about 34% between 2000 and 2020, it has stagnated or worsened in most regions of the world from 2016 to 2020 [2]. Almost 95% of all maternal deaths occurred in low and lower middle-income countries (LMICs) in 2020 [2]. Maternal deaths are often due to obstetric haemorrhage, hypertensive disorders and sepsis, which occur mainly during childbirth [4–6]. To reduce maternal and newborn deaths, there is consensus that women should access good-quality and timely facility-based delivery care by skilled personnel [7, 8]. However, there is wide variation in access to facility-based or skilled births within and across countries [9]. For instance, the proportion of skilled birth attendance deliveries was above 90% in 25 of the 80 LMICs, and below 40% in 11 LMICs [9], and across 25 sub-Saharan African countries, the rate of skilled birth attendance ranged from 24% in Chad to 97% in South Africa [10].

Demand for health care (e.g., facility-based delivery care) is influenced by demand-side and supply-side factors. Demand-side determinants are factors influencing the ability to use health services at individual, household or community levels (e.g., awareness, education, and economic status). Supply-side determinants are aspects inherent to the health system particularly at the point of service delivery (e.g., availability of services, medicines, diagnostics, and medical personnel) which may influence service delivery and uptake by individuals, households or the community [11–13]. Policy makers and practitioners need to understand a holistic picture of the determinants of demand on the demand and supply side, and the relative influence of each, in order to prioritize actions to increase uptake of delivery care services and improve maternal and newborn health outcomes. To date, many studies have examined the demand-side determinants of facility-based delivery care [14–18], with relatively few studies examining supply-side or facility-level determinants [14, 19, 20]. To our knowledge, the demand- and supply-side determinants of facility-based deliveries have not been studied simultaneously, and the relative contributions of each group of factors to demand have not been measured. This represents a significant research gap that warrants investigation. In this case, there is a need to shed light on the comprehensive interplay between the multifaceted demand and supply-side factors, allowing us to construct a more comprehensive and nuanced understanding of the factors associated with utilisation of facility-based delivery care.

This study aimed to broaden the understanding of the factors associated with facility-based delivery care and relative influence of these factors on how they associate with the demand for facility-based delivery care in the Tanzanian context. We used multiple sources of primary data (a survey of patients, households, and facilities) which offers a wider range of demand- and supply-side factors than available through routine surveys (e.g., demographic and health survey (DHS), service provision assessment (SPA)) [21, 22].

## Materials and methods

### Study setting

This study was conducted in three regions of Tanzania: Pwani, Morogoro and Lindi, with the population size of 2 million, 3.2 million, and 1.2 million in 2022, respectively [23]. The study surveyed all seven districts in Pwani, three districts in Morogoro (Morogoro urban, Morogoro rural and Mvomero), and one district in Lindi region (Kilwa). While Tanzania has made substantial progress on child survival, there has been little improvement in maternal health, which stands at 556 deaths per 100,000 live births in 2016 [24, 25]. Although almost all Tanzanian pregnant women accessed at least one antenatal care visit, a smaller share delivered at a facility (63%) and received postnatal care (33%) [24]. Public health facilities are the largest health service provider (70%) in Tanzania, and are organised in a hierarchical administrative structure: dispensaries serve villages, health centres serve wards with multiple villages, and hospitals serve a district or region. Most public health facilities in Tanzania are inadequately funded and experience shortages of staff, drugs and supplies [25–27]. The public health system in Tanzania is financed through general taxation (34%), donor support (36%), out-of-pocket payments (22%), and health insurance contributions (8%) [28]. Tanzania has an exemption and waiver policies to protect poor and vulnerable groups including pregnant women and children [29, 30], but the enforcement of these policies has been weak and exempted patients still incur out-of-pocket payments [31, 32].

#### Conceptual framework

**Fig. 1:**
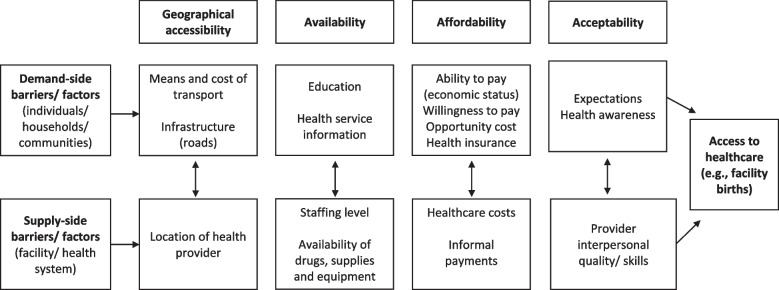
Conceptual framework adapted from Jacobs et al., [11]

We developed a conceptual framework based on the demand and supply-side access barriers presented by Jacobs et al., [11]. They identified four dimensions of access (geographical accessibility, availability, affordability, and acceptability) from previous literature [12, 13, 33–35]. Our framework (Fig. 1) adapted the Jacobs et al. [11] framework by expanding access barriers on the demand and supply side within each dimension as follows: geographical accessibility (location of the health facility, means and cost of transport); availability (drugs, equipment, healthcare workers, information on health services); affordability (medical costs, informal payments, clients’ ability and willingness to pay); and acceptability (provider interpersonal care, individuals expectations, patients assertiveness and level of awareness about health services). Most of the demand-side barriers presented in Jacobs et al., [11] were also consistent with the *Grossman model* reflecting on the human capital model of the demand for health [35, 36] and the *Andersen’s behavioural model* of health care utilisation [34, 37]. The Andersen model includes three groups of individual level factors: (i) predisposing factors like age, household size, education, and parity; (ii) enabling factors like income, and health insurance coverage; and (iii) perceived need factors for healthcare such as health status or illness.

#### Data

We used baseline cross-sectional data that were collected as part of an evaluation of the impact of a pay-for-performance programme in Pwani region, including data collected from two neighbouring comparison regions (Morogoro and Lindi) [38, 39]. A cross-sectional survey of 150 facilities, 1494 patients, and 2846 households was carried out in 11 district councils across the three regions. The 150 facilities included 12 hospitals, 32 health centres and 106 dispensaries as the primary sampling unit; and the majority were public facilities (82%) than non-public facilities. At each facility, we conducted exit interviews with a sample of at most 10 patients after receiving maternal and child health care (antenatal care, postnatal care and selected childhood immunizations) and other outpatient services (malaria, respiratory infection and diarrhoea). From each health facility’s catchment population, 20 eligible households (with women aged 15–49 years who gave birth in the last 12 months) were randomly sampled. All surveys were carried out by trained enumerators from January to February 2012. A facility survey and patient exit interviews captured supply-side factors at facility-level, while a household survey captured demand-side factors at the household and women level. All interviews were conducted in Swahili, and all survey tools were pre-tested for consistency, relevance, and clarity.

#### Outcome

Our primary outcome of interest was place of delivery among women who have given birth 12 months before the survey. This was measured through a household survey as an indicator variable which equals 1 if a woman gave birth in a health facility (institutional delivery) and equals 0 otherwise. We were interested with facility births irrespective of where that facility was located.

#### Explanatory variables

The explanatory variables as potential determinants of a facility-based delivery were selected based on the conceptual framework described above (Fig. 1). The unit of analysis was a woman because the outcome of interest was measured at the woman level. Thus, each supply-side factor measured at the facility level through a facility survey and exit interviews was shared among women residing in a particular facility catchment area. For instance, facility staffing level at *facility A* and average value of all patients from exit interviews (e.g., waiting time) were taken as facility-level factors and linked to all women in the catchment area of *facility A*. All demand-side factors were estimated from the survey of women, with an individual observation for each woman. Only one facility-level factor (facility likelihood of charging delivery fee) was estimated from the survey of women in a facility’s catchment area. This was estimated based on the share of women who paid for delivery care out of the sample women in a facility catchment area.

The *household/ woman-level characteristics* (demand-side factors) included: the woman’s age in years, her education level (4 categories), occupation status (4 categories), religion (Muslim vs. not Muslim), number of births (parity 1 vs. 2+), household wealth status (measured as wealth terciles (poorest, middle, least poor) derived from an index of assets and housing particulars), household ownership of health insurance (binary ‘yes’ / ‘no’), and women experience of care based on antenatal care visits (4 + visits vs. less visits).

The *facility-level characteristics* (supply-side factors) included factors measured through the facility survey across targeted 150 facilities, where targeted households/ women were sampled: facility level of care (hospital and health centre, vs. dispensary), facility ownership (public vs. non-public (private for profit, faith-based)), number of medical staff (doctors, clinicians, nurses, paramedics), number of maternity beds, availability of outreach services in the last 90 days, availability of utilities (water and electricity), availability of drugs for delivery care (an index based on three items: oxytocin, misoprostol, and ergometrine), and facility location (rural/ urban district). Other facility-level characteristics included facility likelihood of charging fees for delivery care measured from the *women survey* (percentage of women who paid out-of-pocket in the catchment area). Additional facility-level characteristics were estimated from *exit interview data* such as average patients’ waiting and consultation time in minutes, average patients’ travel time to access care (in minutes), and interpersonal quality based on patient-providers’ interaction score (unweighted mean score of seven items: *spend enough time with client, given enough information about illness, staff had good explanation, treated with respect, cared more about patients, attended privately without being seen and without being heard*).

#### Statistical analysis

We performed a descriptive analysis of the main outcome of interest as well as individual women, household and facility-level characteristics. To identify the demand and supply-side factors associated with the demand for facility-based delivery care, we used a multilevel modelling technique (mixed-effect logistic regression) to account for the hierarchical structure of the data (because women/households are nested within the catchment area of facilities) [40–42]. The use of multilevel mixed-effect logistic regression, also termed a random effects model [43], assumes that there is greater variation in outcomes between women in the catchment area of different facilities than between women in the catchment area of the same facility, since the experience of care at a given facility is likely to be more uniform than the experience of care at different facilities. To confirm this assumption, two prior analyses were performed. First, we estimated an empty/ random intercept model to determine the random variance and assessed the presence of random intercept variations through the likelihood ratio test. We found variation in variance estimates and the likelihood ratio test for the intercept only model was significant (Chi-square = 105.95, *p*-value < 0.01) (Additional file 1: Appendix Table 1), indicating a significant improvement in fit with random intercepts compared to a standard logistic model (Additional file 1: Appendix Table 1) [40]. Second, we estimated the intraclass correlation (ICC) to capture the degree of facility random effects or between facility variation in the outcome. The ICC results suggest that only 19.6% (ICC = 0.196) of the outcome variation was explained by between facility variation, with the majority being driven by within facility variation (Additional file 1: Appendix Table 1). We therefore used a multilevel mixed-effects model (random effects model) due to the presence of random intercept variations and a significant likelihood ratio test, together with the low intraclass correlation capturing the degree of facility random effects or within facility variation in the outcome (Additional file 1: Appendix Table 1). The following multilevel mixed-effect logistic regression was used to estimate simultaneously both levels of determinants (household/woman and facility).

**Table 1:** Descriptive statistics of dependent and explanatory variables (*N* = 2846)

Variables	n	%	Mean [SD]
**Panel A: Outcome variable**			
Facility-based delivery care	2,443	85.8%	
**Panel B: Demand-side factors**			
Maternal age (in years)	2,846		26.4 [6.6]
Education level (= 1 if no formal education)	565	19.9%	
Education level (= 1 if some primary)	229	8.1%	
Education level (= 1 if primary/ some secondary)	1,844	64.8%	
Education level (= 1 if secondary/ above)	208	7.3%	
Occupation status (= 1 if formal sector worker)	55	1.9%	
Occupation status (= 1 if farmer)	1,435	50.4%	
Occupation status (= 1 if self-employed business)	589	20.7%	
Occupation status (= 1 if other)	767	26.9%	
Religion (= 1 if Muslim)	2,167	76.1%	
Religion (= 1 if non-Muslim)	679	23.9%	
Woman with parity 1 (first birth)	913	32.1%	
Household wealth status (= 1 if poorest Tercile)	956	33.6%	
Household wealth status (= 1 if middle Tercile)	954	33.5%	
Household wealth status (= 1 if least poor Tercile)	936	32.9%	
Women with a health insurance	241	8.5%	
Women with at least four antenatal care visits	1,919	67.4%	
**Panel C: Supply-side factors**			
Availability of utilities (= 1 if water & electricity available)	1,525	53.6%	
Facility outreach services (= 1 if conducted in last 90 days)	2,174	76.4%	
Facility location (= 1 if rural district)	2,329	81.8%	
Facility level of care (= 1 if dispensary)	1,980	69.6%	
Facility ownership (= 1 if public owned)	2,376	83.5%	
Number of maternity beds [SD]	2,846		3.5 [4.5]
Number of medical staff (clinicians, nurses, paramedics)	2,846		15.6 [30.4]
Facility average waiting time (minutes)	2,846		44.2 [29.3]
Facility average consultation time (minutes)	2,846		12.9 [5.2]
Patients’ travel time from home to facility (minutes)	2,846		30.1 [15.6]
Availability of delivery drugs (oxytocic)(index)	2,846		0.44 [0.27]
Facility average score in interpersonal quality	2,846		0.79 [0.11]
Facility status of charging fee for delivery care	2,846		0.21 [0.19]

*SD* standard deviation; median staffing level (5); mean staffing level for dispensaries (mean 5, median 3), health centres (mean 22, median 18), hospitals (mean 90, median 76)

$$\mathrm{Logit}\;\{\mathrm{pr}(Y_{ij})\}=\mathrm\alpha+\beta_{\mathit1}\mathit\;X_{ij}+\beta_{\mathit2}Z_j+\mu_j+{\mathrm\varepsilon}_{\mathrm{ij}},$$

where *i* and *j* are individual women and health facilities, respectively. ${Y}_{ij}$ is a binary indicator for the outcome of interest (i.e., place of delivery); ${X}_{ij}$ represents individual and household covariates, ${Z}_{j}$ represents health facility level covariates, ${\mu }_{j}$ represents random intercepts for facilities ${\mu }_{j}$. ${\beta }_{1}$ and ${\beta }_{2}$ are regression coefficients, α is a constant term and the error term is ${\epsilon }_{ij}$. As a robustness check, we also estimated a reduced model by using logistic backward stepwise regression (Additional file 1: Appendix Table 2). All analyses were performed using STATA software version 16.

**Table 2:** Factors associated with utilisation of facility-based delivery care (*N* = 2846)

	Mixed-effects model	
Determinants	Odds Ratio (OR)	[95% CI]
**Demand-side factors**		
Maternal age (in years)	1.01	[0.99 to 1.03]
Education level		
No education (ref.)	1.00	
Some primary	1.46*	[0.95 to 2.25]
Primary/ some secondary	1.74***	[1.32 to 2.29]
Secondary or above	4.13***	[1.65 to 10.3]
Occupation status		
Formal sector worker (ref.)	1.00	
Farmer	0.31	[0.04 to 2.48]
Self-employed business	0.61	[0.07 to 4.98]
Other	0.38	[0.05 to 3.06]
Religion type		
Non-Muslim (ref.)	1.00	
Muslim	1.36**	[1.01 to 1.84]
Parity/ number of births		
At least 2 births (ref.)	1.00	
Woman with parity 1 (1st delivery)	1.72***	[1.23 to 2.41]
Household wealth status		
Poorest tercile (ref.)	1.00	
Middle tercile	0.97	[0.74 to 1.27]
Least poor tercile	1.93***	[1.32 to 2.81]
Health insurance		
No health insurance (ref.)	1.00	
With health insurance	0.73	[0.46 to 1.14]
Antenatal care visits		
< 4 visits	1.00	
≥4 visits	1.33**	[1.05 to 1.70]
**Supply-side factors**		
Availability of utilities		
Available water & electricity	1.16	[0.80 to 1.68]
No water & electricity (ref.)	1.00	
Facility conducted outreach services in last 90 days		
Yes	1.55**	[1.05 to 2.28]
No (ref)	1.00	
Facility location		
Rural district	1.24	[0.76 to 2.02]
Urban district (ref.)	1.00	
Facility level of care		
Dispensary	0.92	[0.56 to 1.50]
Health centre/ hospital (ref.)		
Facility ownership		
Public owned	0.90	[0.53 to 1.53]
Non-public (ref.)		
Number of maternity beds	1.01	[0.96 to 1.06]
Number of medical staff (clinicians, nurses, paramedics)	1.00	[0.99 to 1.01]
Facility average waiting time (minutes)	0.99*	[0.99 to 1.00]
Facility average consultation time (minutes)	1.04**	[1.01 to 1.07]
Patients’ travel time from home to facility (minutes)	0.99**	[0.98 to 0.99]
Availability of delivery drugs (oxytocic)(index)	1.16	[0.61 to 2.20]
Facility average score in interpersonal quality	5.11**	[1.12 to 23.4]
Facility status of charging fee for delivery care	0.24***	[0.09 to 0.63]
Constant	1.20	[0.08 to 17.9]
**Random effects parameters**		
Facility random variance (SE)	0.41 (0.11)	
Likelihood ratio test (mixed-effects vs. logit model),${\chi }^{2}$	36.2***	

*Ref* reference categories as indicated in parentheses; * *p* < 0.10, ** *p* < 0.05, *** *p* < 0.01

## Results

### Descriptive statistics

The rate of facility-based deliveries among our sample of 2846 women giving birth in the 12 months preceding the survey was 85.8% (Table 1). Women were aged 26 years old on average, and most were educated to the primary level and above. The majority of women were farmers (50.4%), Muslim (76.1%), without health insurance (91.5%), had attended at least four ANC visits (67.4%), and had given birth more than once (67.9%).

Most facilities had both water and electricity (54%), conducted outreach services (76%), were located in rural districts (82%), were dispensaries (70%), and publicly owned (84%) (Table 1). On average, facilities had three maternity beds and sixteen medical staffs, with median values of two maternity beds, and five medical staffs. The surveyed facilities had low availability of delivery drugs (oxytocic) at 44% on average. According to patients who accessed care in these facilities, they spent on average 30 min travelling from home to the facility, 44 min waiting for care, and 13 min in consultation with a provider. Patients rated providers with an average interpersonal quality score of 79%, and the average share of women who paid for delivery care across facilities was low (21%).

### Factors influencing facility-based delivery

Women who were educated, Muslim, given birth only once, wealthier, and had at least four ANC visits, were more likely to deliver in a health facility than their counterpart women (Table 2). Specifically, women’s level of education associated positively and significant with increased demand for facility-based delivery. For instance, women with secondary education/ above were almost 4 times more likely to have facility births [OR = 4.13 (CI: 1.65 to 10.3)] compare to women with no education. Muslim women were 1.4 more likely to have facility-based delivery [OR = 1.36 (CI: 1.01 to 1.84)] compared to non-Muslim women. Women who delivered for the first time were 1.7 more likely to deliver in a facility [OR = 1.72 (CI: 1.23 to 2.41)] compared to those with at least two births/ higher parity. The least poor (wealthier) women were almost twice as likely as poorest women to have facility-based delivery [OR = 1.93 (CI: 1.32 to 2.81)]. Women with at least four ANC visits during pregnancy were 1.3 more likely to have facility births [OR = 1.33 (CI: 1.05 to 1.70)] compared to women with less than four ANC visits.

Facility-based deliveries were more likely for women living near facilities offering outreach services, longer consultation times and offering higher interpersonal quality. Women living near facilities offering outreach services were almost twice as likely as women living near facilities without outreach services to deliver in a facility [OR = 1.55 (CI: 1.05 to 2.28)]. The odds of a facility-based delivery increased by 4% [OR = 1.04 (CI: 1.01 to 1.07)] for every additional minute of consultation time for facility outpatients; and, for each unit increase in facility-level interpersonal quality score, the odds of facility-based delivery increased five times on average [OR = 5.11 (CI: 1.12 to 23.4)].

On the other hand, longer waiting times, longer travel time to reach the facility, and a higher chance of charging delivery fee significantly associated with the reduced demand for facility-based delivery. The odds of facility-based delivery were reduced by 1% for each additional minute of travel time to health facility [OR = 0.99 (CI: 0.98 to 0.99)] and for each additional minute of waiting time for outpatient care [OR = 0.99 (CI: 0.98 to 1.00)]. For each percent increase in charging delivery fee at the facility associated with the reduced odds of facility-based delivery by 76% on average [OR = 0.24 (CI: 0.09 to 0.63)].

Overall, demand-side factors had stronger association with the demand for facility-based delivery care than supply-side factors (Table 3). The demand side factors explaining 79% of demand for facility-based delivery care, while the remaining share was explained by supply-side factors.

**Table 3:** Relative contributions of demand- and supply-side factors on demand using the partial log likelihood method

Factors	Log likelihood (If specific group factors excluded)	Partial effect (change in log likelihood)	Relative effect (% of sum of change in log likelihood)	Order of importance
Full model (mixed-effects)	–1030.8098	–		
Demand-side factors	–1088.0576	–57.2478	78.6%	1
Supply-side factors	–1046.4232	–15.6134	21.4%	2

The reduced model revealed almost similar significant factors associated with the demand for facility-based delivery (Additional file 1: Appendix Table 2). The only discrepancy was the addition of staffing level which became statistically significant in a reduced model, while wealth status and religion were dropped.

## Discussion

We estimated the proportion of women delivering in a health facility and identified the demand and supply-side factors associated with women’s decision regarding place of delivery across three regions in Tanzania. We found 86% of women delivered in a health facility with demand for facility-based delivery being higher for women who were educated, Muslim, wealthier, with first birth, and those who had at least four antenatal care visits. In terms of supply-side factors, facility births were more likely for women living near facilities offering outreach services, longer outpatient consultation times and higher interpersonal quality, while few facility births happened in communities that were far from facilities, or where the nearest facility had longer waiting time, and charged delivery fees. The type of facility by ownership and level of care, as well as the staffing level and availability of drugs did not associate with women’s decision on place of delivery. Overall, demand-side factors had relatively stronger association with the demand for facility births by 79% compared to supply-side factors.

The coverage of facility-based delivery was higher than average national coverage of 63% reported in the 2015/16 Tanzania DHS [24]. Our findings regarding the demand-side determinants of facility-based delivery are consistent with demand-side determinants of maternal health care utilisation (including delivery care) in the wider literature from LMICs [14–19], which found increased facility-based delivery among women who were educated [15–17, 19], Muslim [15], wealthier [15–17, 19], with low parity [15], and had higher utilisation of antenatal care [16, 17, 44]. Higher education level and economic status associated with higher demand for facility births partly because they both serve as enabling factors in healthcare seeking by increasing awareness and reducing cost barriers [11, 36, 45, 46]. Low parity associated with higher demand for facility births partly because of the absence of prior experience of delivery which pushes women to seek facility-based care as a way to avoid unforeseen health risks or complications. Many antenatal care visits associated with high demand for facility birth because healthcare workers use the antenatal care platform to encourage women for facility-based delivery care [47, 48]. The religion factor may remain context-specific; however, other studies have also found Muslim women utilised facility-based delivery care more than Christians in Malawi [49] and Tanzania [50].

The identified supply-side factors influencing facility-based delivery concur with findings from other studies. For instance, some studies reported that higher coverage of facility births was associated with facilities offering outreach services [51], facilities with longer consultation time and higher interpersonal quality [52]. Also, fewer facility births were associated with longer waiting time [53–55], longer travel time [56–58], and higher incidences of paying for delivery care [56, 57, 59, 60]. However, these studies did not examine both demand- and supply side factors simultaneously nor established the relative contribution of each group of factors, contrary to our analysis. The demand for facility births associated positively with increased outreach services, high interpersonal quality, and consultation time possibly because these factors reflect increased providers’ responsiveness to clients and patients’ satisfaction [61]. Direct healthcare payments (e.g., out-of-pocket payments) and indirect costs (e.g., travel and waiting time) were associated with low demand for facility births; this is possibly because they are access barriers which deters health care access and demand [12, 62].

This study expands the understanding of factors affecting healthcare demand in the following aspects. First, our study is the first study to examine simultaneously the demand and the supply-side factors associated with the demand for health care particularly facility delivery care. Second, this study is the first to quantify the relative contributions of demand- and supply-side factors in associating with demand for health care and more specifically on facility delivery care. Third, this study used primary data offering a wider range of supply-side factors from linking multiple data sources (patients, household, facility) (e.g., patient level information such interpersonal quality, waiting and consultation times). It is therefore important for routine facility surveys such as SPA to collect patient level information at the point of service delivery.

Our study had some limitations. First, we relied on only 20 women from each facility’s catchment area to estimate the rate of facility-based delivery and ascertain associated demand-side determinants. This may not reflect the actual status of all reproductive women in the catchment area. Second, we used women’s nearest facility to characterise the supply-side determinants, but due to bypassing tendency especially for primary healthcare facility in Tanzania [63], not all sampled women delivered in their nearest or local facility [64]. Nevertheless, this study is interested in how the characteristics of a woman’s local facility shapes the demand for delivery care. In other words, it is still reasonable to assume the local facility characteristics may influence the decision on where to deliver even if they do not delivery at their local facility. It is also important to know that some bypassing events are being referred from their local facilities. Third, facility level characteristics measured at the time of the survey, may not necessarily reflect the exact status of the facility prior to or at the time a woman delivered at health facility. Fourth, we were unable to include all potential determinants of demand such as actual distance from home to health facility, previous experience of delivery care, influence of women social network, facility capacity limit and traditional birth attendants. We used proxy measures such as travel time in minutes for distance, however, this relied on patient recall which may not have been accurate. Fifth, we used data from only three regions, which may limit the generalisability to other parts of the country, however, there was substantial variation within those three regions. Sixth, we used data from surveys of 2012, since such surveys captured comprehensive data for demand- and supply-side factors from multiple sources (patients, households, facilities) which are lacking in more recent surveys (e.g., DHS and SPA). It is possible some access barriers have been addressed that existed previously (e.g., construction of more facilities should reduce travel time, more resources at facilities might reduce charging delivery fees and drug stock-outs). Lastly, some facility characteristics were measured from a sample of patients interviews during the survey (e.g., waiting and consultation time, interpersonal quality), which may not necessarily represent the true characteristics of the facility in the presence of all patients.

We highlight important implications for improving demand for facility-based delivery care services in Tanzania. The predominance of demand-side influences indicates the need for targeted health education programmes in the community to improve awareness on care seeking among those with lower levels of education and higher parity. Lower demand among poorer households together with the large effect of delivery care costs on decision making, highlight the importance of interventions to increase the affordability of care especially for poorer groups (e.g., strengthening health insurance coverage and exemptions). The lack of effect of health insurance on demand, suggests that the benefit package may not be sufficient to offset financial barriers beyond user charges [65]. It is something to be considered by policy makers when designing the mandatory universal health insurance in Tanzania. Also, additional financial resources may be needed to improve facility level activities including offering outreach services [66, 67].

Moreover, we underscore the importance of antenatal care as a channel through which to encourage facility-based deliveries, suggesting that policy initiatives that enhance access to regular antenatal care are likely to increase the rate of institutional deliveries. Equally, our study highlights that local facility reputation characterised in our study by the length of outpatient consultations and the quality of patient-provider interactions has a substantial influence over decision-making for where a women would go for delivery. For instance, poor quality of services at local or nearest facility influences clients to bypass towards other or even a higher-level facility [63, 64]. In this case, policy makers need to incentivise health workers to improve productivity, motivation and provision of quality health care, for instance, by providing timely benefits (e.g., houses, extra-duty allowances), remuneration and training [68, 69]. Strengthening service provision is important because quality of care provided –including interpersonal quality such as respectful care –is critical to increase the demand for facility-based maternity care [52, 70–73]. To increase healthcare demand, policy makers should also ensure adequate supply of health workers to increase consultation times, reduce waiting time [74], and adequate development of primary health facilities to reduce both travel and waiting time [75]. Policy makers should think about additional ways to reduce the travel time such as improving road and public transport infrastructure [76]. However, the success of this approach depends on other sectors beyond the health sector (e.g., transportation and infrastructure sector), which indicates the need for multisectoral approaches to reduce access/ geographical barriers [65]. For effective results, there is a need for multifaceted interventions to enhance demand [69].

## Conclusion

This study shows the importance of assessing both demand- and supply-side factors associated with the demand for facility-based delivery care in order to inform evidence-based interventions. Interestingly, we found the demand for facility-based delivery care was influenced more by demand-side factors than supply-side factors. This finding reinforces the need for policy makers to design interventions or strategies to improve healthcare demand like health education to raise awareness towards care seeking among less educated groups and those with higher parity, reduce financial barriers to access (including time costs to reach and access care), and policy interventions to enhance interpersonal quality in service provision.

### Supplementary Information

**Additional file 1: Appendix Table 1. **Random effects parameters for random intercept only model (empty model).** Appendix Table 2. **Reduced model by using logistic backward stepwise regression.

## Data Availability

The data have been uploaded into a data repository. The DOI URL for the dataset is: 10.5281/zenodo.21709.

## Abbreviations

ANC: Antenatal Care
CI: Confidence Interval
DHS: Demographic Health Survey
ICC: Intraclass Correlation
LMICs: Low- and middle-income countries
MMR: Maternal Mortality Rate
OR: Odds Ratio
SD: Standard Deviation
SDG: Sustainable Development Goal
SPA: Service Provision Assessment
