# Supplementary material for: Supply-side factors influencing demand for facility-based delivery in Tanzania: a multilevel analysis
Source: Health Econ Rev. 2023 Nov 6;13:52. doi: 10.1186/s13561-023-00468-1 (PMC10629065; doi:10.1186/s13561-023-00468-1)
Supplement: Supplementary file 1 — Additional file 1: Appendix Table 1. Random effects parameters for random intercept only model (empty model). Appendix Table 2. Reduced model by using logistic backward stepwise regression. [file 13561_2023_468_MOESM1_ESM.docx]

**Appendix Table 1: Random effects parameters for random intercept only model (empty model)**

| **Facility random effects** | **Institutional delivery** |
| --- | --- |
| Random variance (SE) | 0.803 (0.169) |
| Intraclass correlation (ICC) | 0.196 |
| Likelihood ratio test, $\chi^{2}$ (p-value) | 105.95 (p<0.001) |

Notes: SE=Standard Error

**Appendix Table 2: Reduced model by using logistic backward stepwise regression**

| **Determinants** | **Odds Ratio** | **Coefficient** | **z-value** | **P-value** |
| --- | --- | --- | --- | --- |
| **Demand-side factors** |  |  |  |  |
| Education level (=1 if no formal education) (ref.) |  |  |  |  |
| Education level (=1 if some primary) | 1.495 | 0.402 | 1.94 | 0.053 |
| Education level (=1 if primary/ some secondary) | 1.821 | 0.599 | 4.68 | 0.000 |
| Education level (=1 if secondary/ above) | 6.925 | 1.935 | 4.47 | 0.000 |
| Woman with parity 1 (first delivery) | 1.604 | 0.472 | 3.41 | 0.001 |
| At least four antenatal care visits | 1.399 | 0.336 | 2.94 | 0.003 |
|  |  |  |  |  |
| **Supply-side factors** |  |  |  |  |
| Staffing level (medical staff) | 1.012 | 0.012 | 3.56 | 0.000 |
| Facility conducted outreach services in last 30 days | 1.453 | 0.374 | 2.88 | 0.004 |
| Waiting time in minutes | 0.994 | -0.006 | -3.00 | 0.003 |
| Consultation time in minutes | 1.033 | 0.032 | 2.71 | 0.007 |
| Interpersonal quality score | 4.482 | 1.500 | 2.90 | 0.004 |
| Facility charging delivery fee | 0.319 | -1.142 | -3.98 | 0.000 |
| Travel time to facility in minutes | 0.988 | -0.012 | -3.69 | 0.000 |
| Constant | 0.881 | -0.126 | -0.26 | 0.793 |
